# Metal organic framework functionalized reflective tapered fibre-optic surface plasmon resonance based refractometer for vapor sensing

**DOI:** 10.1038/s41598-026-61422-9

**Published:** 2026-07-07

**Authors:** Akila Chithravel, Naveen Ilango, Jain Jacob, Jithin John, Lakkakula Satish, Sandeep Munjal, Sruthi P. Usha, Anand M. Shrivastav

**Affiliations:** 1 Optics and Nano Electronics Laboratory, Department of Physics and Nanotechnology, College of Engineering and Technology, SRM Institute of Science and Technology, Kattankulathur, Tamil Nadu 603203 India; 2 Faculty of Engineering, Christian-Albrechts-Universität zu Kiel (Kiel University), 24143 Kiel, Germany; 3 Marine Natural Products and Bio-Polymers Division, CSIR - Central Salt & Marine Chemicals Research, Bhavnagar, Gujarat 364001 India; 4 National Forensic Sciences University, Gandhinagar, Gujarat 382007 India

**Keywords:** Tapered fiber optic sensor, Reflective tip probe, Surface plasmon resonance, Metal organic frameworks, ZIF-8, Plasmonics, Materials science, Optics and photonics

## Abstract

Metal-organic frameworks (MOFs), such as ZIF-8, have been extensively studied for their gas adsorption and chemical sensing capabilities owing to their high surface area, porosity, and tunable host-guest interactions. The adsorption of a guest molecule modifies the effective refractive index of ZIF-8 through pore-filling effects, rendering it appropriate for optical sensing. In the present study, we investigate the design of a reflective tip-based tapered fiber optic surface plasmon resonance (FO-SPR) platform functionalized with a ZIF-8 absorbing layer for the detection of chemical vapors. Experimentally measured, analyte-dependent refractive index changes of ZIF-8 are integrated into a Transfer Matrix Method (TMM) based reflective tapered FO-SPR model to systematically evaluate the influence of taper ratio on resonance wavelength, sensitivity, spectral broadening, and figure of merit for different vapor analytes. The analyte-dependent response towards tetrahydrofuran (THF), ethanol, and dimethylformamide (DMF) is analysed in terms of the refractometric modulation characteristics of ZIF-8 using the SPR transduction mechanism. To the best of our knowledge, this study constitutes the first theoretical investigation of MOF pore-filling dynamics within a reflective-tapered FO-SPR configuration. The sensor performance enhances significantly as the taper diameter decreases from 600 to 200 μm with sensitivity increasing from 9.38 to 10.76 nm/Volume Factor for THF and from 4.83 to 5.69 nm/Volume Factor for DMF. The operating range of the sensor is governed by the effective refractive index tunability of the ZIF-8 layer, which varies from 1.355 to 1.421, ensuring conditions necessary for SPR excitation relative to the fiber core. These results establish a fundamental framework for optical refractometric vapour sensing and provide practical design guidelines for MOF-functionalized tapered FO-SPR platforms.

**Supplementary Information:**

The online version contains supplementary material available at 10.1038/s41598-026-61422-9.

## Introduction

Over the past two decades, surface plasmon resonance (SPR) has emerged as a leading technique for optical refractometric sensing of chemical and biological analytes in complex environments^1,2^. Optical fibers are particularly well suited for such applications due to their compact and lightweight design, mechanical flexibility, ease of integration, and ability to transmit high-quality light with minimal losses over long distances, which render them ideal for remote sensing and in-situ monitoring^3–7^. In reflective tip fiber configurations, guided light undergoes total internal reflection (TIR) as it travels to the fiber tip and is then reflected through the same fiber core. Such a design effectively doubles the interaction between light and the sensing medium, thereby enhancing sensitivity^6,8,9^. Furthermore, the reflective tip design simplifies the setup by eliminating the need for complex external optical components at the sensing location, improving practicality for point-of-care and portable sensing applications. Since measurement occurs at the fiber tip, the probe can be extended to remote, hazardous, or otherwise inaccessible areas while the detection equipment remains safely positioned elsewhere, enabling real-time contactless monitoring.

Integrating a tapered fiber configuration with a tip probe significantly enhances refractive index (RI) sensitivity, as reducing the fiber core diameter increases the evanescent field penetration into the sensing region^7,10–13^. This facilitates strong plasmonic generation, allowing the detection of small changes in the RI of the surrounding medium through their interactions, making it suitable for small-volume analysis. In 2013, Yaun et al. reported a theoretical study on a tapered fiber optic SPR tip probe for RI sensing^8^, focusing on the influence of geometrical parameters of the probe on the sensor’s performance, like resonance wavelength and sensitivity. The study reported that increasing the tip angle induces a red shift in the resonance wavelength and broadens the SPR curve. In a subsequent study, Lu et al. developed an optical fiber RI sensor using a long-tapered tip, generating a circular interference pattern that exhibits high sensitivity to environmental RI variations^9^. Although their image-based evaluation method achieved good sensitivity, it was limited by a relatively narrow dynamic RI range. In contrast, when the sensor was characterized using back-reflection with a broadband light source and a spectral analyzer, the sensitivity was reduced because it critically depends on accurately identifying the wavelength region that offers the highest sensitivity.

The recent advances in SPR-based optical fiber sensors have focused on enhancing sensitivity through novel waveguide geometries, plasmonic materials and functional coatings. For example, Ali et al.^14^ demonstrated that a D-shaped optical fiber SPR sensor coated with a double perovskite oxide (DPO) recognition layer over a silver film improves the maximum sensitivity of 6786 nm/RIU. Similarly, an arc-shaped optical fiber SPR sensor functionalized with Ba_2_FeMnO_6_ and LaFeMnO_6_ with DPO coatings achieved 7209 nm/RIU^15^. In addition to advanced dielectric-coated fiber SPR structures, recent efforts have explored the integration of porous MOF materials with plasmonic sensing platforms. Recently, Estany-Macia et al. reported ZIF-8-based SPR and Fabry-Perot sensors for volatile organic compounds (VOC) detection, while Vandezande et al. demonstrated MOF-compatible back-reflection FO-SPR architectures for full-spectrum analysis^16,17^. These studies highlight the potential of advanced dielectric coatings and engineered fiber geometries for improving SPR performance. However, while significant progress has been achieved in enhancing refractive index sensitivity, achieving adequate analyte specificity remains a major challenge for SPR-based fibre optic sensors. To overcome this limitation, the sensor probes are typically functionalized with a recognition layer to enrich the local analyte concentration. Shabaneh et al. demonstrated this approach by coating a tapered fiber tip with graphene oxide nanostructures for aqueous ethanol detection^18^. The high surface area-to-volume ratio of graphene oxide enabled efficient chemisorption of ethanol molecules, significantly affecting the reflectance signal. Similarly, incorporating porous materials such as metal-organic frameworks (MOFs) further enhances this effect by increasing local analyte concentrations within their nanoscale pores^6,19–22^.

Salehifar et al. have reported a theoretical study computing the percentage change in the index of refraction of MOFs (ZIF-7, ZIF-8, ZIF-90, MIL-101(Cr) and HKUST-1) upon exposure to ethanol at different partial pressures, providing a proof-of-concept for VOC detection in lower levels using the RI property of MOF^20^. Furthermore, Kim et al. reported a sensing strategy that exploits small molecule-induced RI changes in MOF thin films by monitoring characteristic absorption features with an optical fiber waveguide platform^23^. Their experimental work demonstrated ZIF-8 and Co/ZIF-8 layers deposited over an etched optical core at the center of a straight fiber, achieving selectivity and rapid response times (< 10 s) for CO_2_ detection, aided by ZIF-8’s hydrophobic nature and porous structure. A thickness of 200 nm was found to yield the maximum response in CO_2_ sensing.

MOF offers structural porosity that supports both physisorption and selective chemisorption. However, the effective RI of MOF and its uniformity of deposition over the fiber core are critical for sensor performance. It has been reported that MOF’s RI varies significantly with guest molecule adsorption, humidity, and the degree of pore filling, with changes of up to 28% depending on the analyte and its concentration^24,25^. Among MOFs, zeolitic imidazolate frameworks (ZIFs) have attracted particular interest for detecting chemical solvents and vapors due to their small pore size and chemical robustness^26–29^. Keppler et al. previously demonstrated the use of ZIF-8, composed of zinc ions linked by 2-methyl imidazolate, as a host material capable of loading various chemical substances, enabling controlled modulation of its RI^21^. By fabricating crack-free ZIF-8 thin films and exploiting pore-filling effects to tune the dielectric properties, they achieved substantial RI modulation suitable for using in integrated optical devices.

Building on these earlier studies, we present a theoretical model for a tapered SPR fiber tip configuration that enables comprehensive spectral analysis and addresses the limitations often associated with experimental back-reflection methods in optical fibers, particularly finding the optical wavelength regime for analysis. The model exploits the pore-filling capability of ZIF-8 to enable accurate identification of the resonance wavelength as a function of analyte identity and volume fraction within ZIF-8. ZIF-8 features moderate porous cavities (~ 12 Å) and smaller apertures (~ 3.4 Å), with an effective aperture window of about 7 Å, enabling selective uptake and encapsulation of guest molecules^30^. This degree of tunability represents a significant advantage over many conventional porous materials. Furthermore, the hydrophobic nature of ZIF-8 prevents encapsulated species from dissolving in aqueous environments, thereby improving sensor reproducibility and long-term durability.

**Fig. 1:**
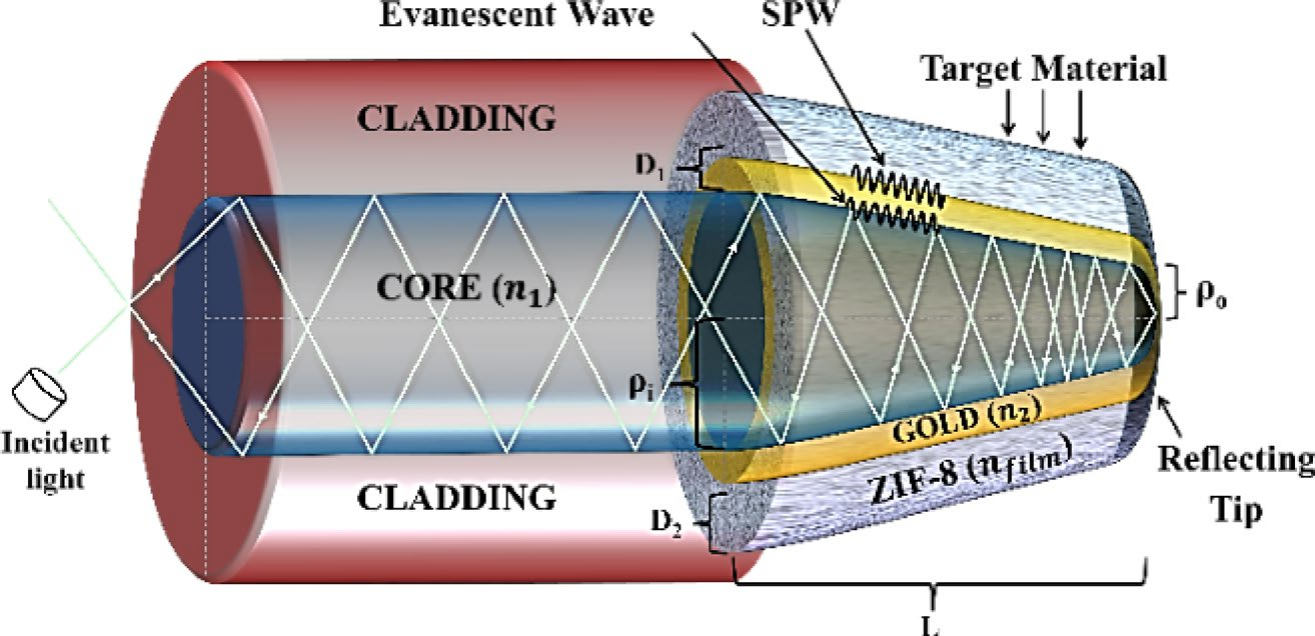
Tapered profile of the fiber optic - SPR tip probe.

Compared with other porous materials, ZIF-8 can encapsulate a broad range of species, including nanoparticles, biomolecules, and organic/inorganic molecules, through its in-situ strategies, with homogeneous distribution and retention of the functional properties of both host and guest^16^. By contrast, non-porous semiconductor metal oxides (SMOs), which are often used for similar purposes, generally require elevated operating temperatures (140–400 °C). In contrast, ZIF-8-based composites can operate effectively at lower temperatures, also when combined with carbon additives, which further decrease energy consumption and mitigate the risk of material degradation. In light of these advantages, we integrate a ZIF-8 functional layer with a fiber optic SPR tapered tip, as illustrated in Fig. 1.

In this work, we first optimize an untapered SPR fiber tip probe model, utilizing gold as the plasmonic transduction layer, followed by a fixed 200 nm thick ZIF-8 sensing layer. Further, the probe is designed to determine the optimal thickness of the gold (Au) metal layer, that maximizes the reflectance. The optimized parameters are then used to guide a detailed numerical analysis of a tapered tip Au-MOF SPR configuration. In this design, the pore-filling effect within the MOF is exploited to evaluate the sensor’s ability to accurately distinguish between different gases and to quantify their presence inside the ZIF-8 pores based on induced spectral changes. Finally, the supplementary information has a proof-of-concept validation of the underlying Au/ZIF-8 SPR sensing mechanism. We demonstrate a reflection-based untapered tip FO-SPR probe functionalized with synthesized ZIF-8 for vapor sensing. The experimental study serves as a baseline demonstration provided in the supplementary information, while experimental realization of the proposed tapered configuration is reserved for future work.

## Theoretical model

The schematic of the proposed tapered tip of the optical fiber sensor configuration is shown in Fig. 1. The sensor probe model follows the conventional Kretschmann configuration with a three-layer structure: a silica core with a diameter of 600 μm, a 35 nm thick Au plasmonic layer and a 200 nm thick ZIF-8 sensing layer in the unclad sensing regime^7,31^. The reflected spectrum for the designed probe is computed using an N-layer model for a multi-layer structure, which requires the thickness and dispersion properties of each layer. The following section describes the RI for optical fiber core, gold and ZIF-8 films, along with the implementation of the N-layer model simulations.

### First layer—optical fiber core

Light is, assumed, initially launched into a step-index multimode plastic-clad silica fiber, the dispersion relation of which is governed by the Sellmeier relation^7,29^:

1 $$\:{n}_{1}\left(\lambda\:\right)=\sqrt{1+\frac{{a}_{1}{\lambda\:}^{2}}{{\lambda\:}^{2}-{b}_{1}^{2}}+\frac{{a}_{2}{\lambda\:}^{2}}{{\lambda\:}^{2}-{b}_{2}^{2}}+\frac{{a}_{3}{\lambda\:}^{2}}{{\lambda\:}^{2}-{b}_{3}^{2}}}$$

where a_1_, a_2_, a_3_, b_1_, b_2_ and b_3_ are the Sellmeier coefficients and λ (µm) is the incident wavelength. The coefficients used for silica are a_1_ =0.6961663, a_2_ = 0.4079426, a_3_ = 0.8974794, b_1_ = 0.0684043, b_2_ = 0.1162414, and b_3_ = 9.896161.

### Second layer—Gold (Au)

For the Au layer, the dispersion relation is described by the Drude model, expressed as:

2 $$\:{n}_{2}\left(\lambda\:\right)=\sqrt{{\epsilon\:}_{m}\left(\lambda\:\right)}=\:\sqrt{{\epsilon\:}_{mr}+i{\epsilon\:}_{mi}}=\sqrt{1-\frac{{\lambda\:}^{2}{\lambda\:}_{c}}{{\lambda\:}_{p}^{2}\left({\lambda\:}_{c}+i\lambda\:\right)}}$$

where λ_p_ and λ_c_ denote the plasma wavelength and collision wavelength, having values 1.6826 × 10^− 7^ m and 8.9342 × 10^− 6^ m respectively.

### Third layer—ZIF-8

**Fig. 2:**
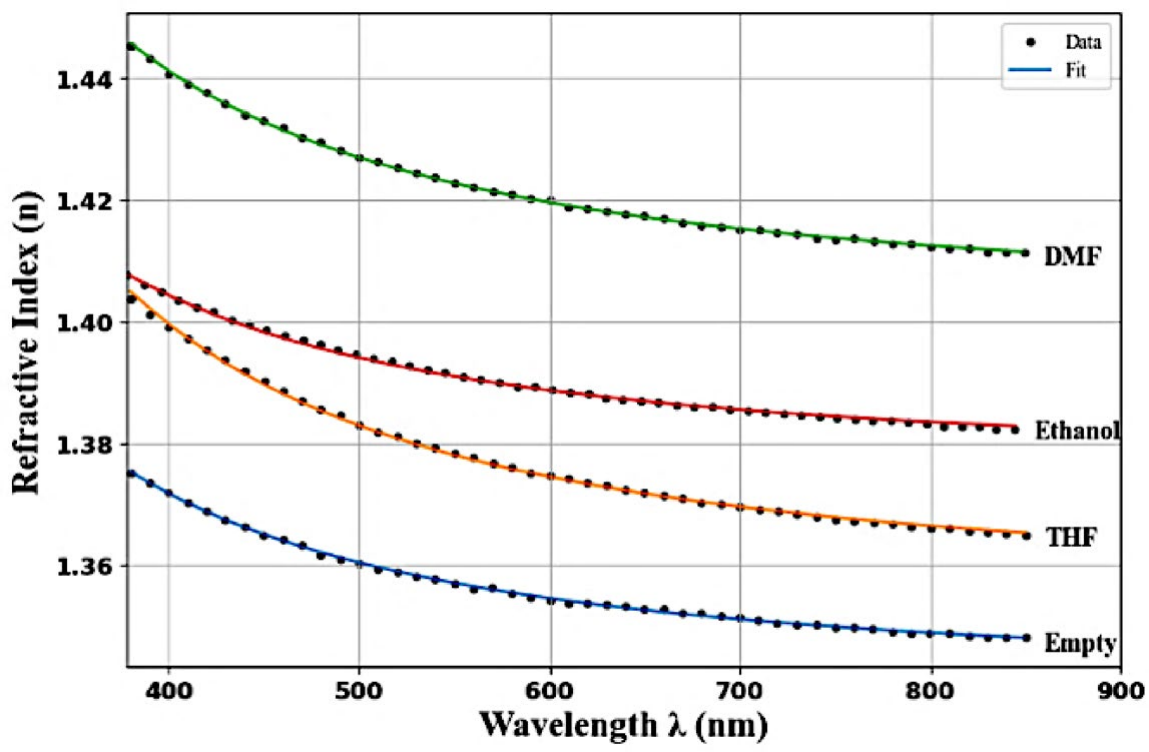
RI dispersion curves for ZIF-8 without and with THF, ethanol and DMF. Reproduced with permission from RSC^21^.

At the end, the sensor structure is capped by ZIF-8 MOF film that serves as the sensing layer, as illustrated in Fig. 1. The effective RI of ZIF-8 changes upon interaction with different gases/chemical vapors due to guest-molecule adsorption within its pores. Determining the RI of ZIF-8 thin films is very challenging due to their porous architecture and dynamic response to guest gas molecules. Hence, to ensure consistency with experimental observations, we adopt the dispersion relation of ZIF-8 from previously reported experimental studies^21^. Keppler et al. demonstrated that ZIF-8 can exhibit significant RI modulations in the visible electromagnetic spectral range, making it suitable for optical devices such as sensors. They fabricated a crack-free ZIF-8 thin film directly on silicon wafers using three deposition cycles of a nanoparticle ZIF-8 precursor solution, adapting a synthesis protocol previously reported by Lu and Hupp^32^. For these MOF films, the effective RI is modelled as a volume-weight average of the indices corresponding to the MOF (*n*_*MOF*_ > 1) and the MOF void cavities (vacuum, *n*_*vacc*_ = 1) and is given by:

3 $$\:{n}_{film}=\sqrt{{f}_{MOF}{{n}_{MOF}}^{2}+{f}_{guest}{{n}_{guest}}^{2}+\left(1-{f}_{MOF}-{f}_{guest}\right){{n}_{vacc}}^{2}}$$

where *f*_*MOF*_ and *f*_*guest*_ are the volume fractions of the MOF and chemical vapors respectively, in the system. Pore-filling or absorption of the guest vapor molecules (with refractive index, n _guest_>1) into the microporous ZIF-8 displaces the vacuum-like cavities and increases the effective refractive index of the MOF layer. Figure 2 shows the change in the RI of the ZIF-8 film with and without loading of guest vapor analyte molecules such as tetrahydrofuran (THF), ethanol and dimethylformamide (DMF), adapted from experimental data reported by Keppler et al.^17^. Each of these vapors has distinct applications and safety implications: THF is widely used in pharmaceuticals, adhesives, and polymer manufacturing, where leakage poses health and safety risks; ethanol detection is essential in breathalyzers, food and beverage production, and biofuel fermentation; DMF is a toxic solvent used in textiles, electronics, and pharmaceuticals, requiring strict exposure limits (typically < 10 ppm). For instance, a ZIF-8 functionalized fiber sensor could therefore be used to monitor DMF levels in factory air vents or worker PPE, warning of hazardous concentrations^21^.

The data points in Fig. 2 are extracted with the open-source tool - https://apps.automeris.io/wpd4/ and fitted with a single oscillator model expressed as^22^:

4 $${n}^{2}-1=\frac{{E}_{d}{E}_{o}}{{E}_{o}^{2}-{\left(E\right)}^{2}}$$

where E, Ed and Eo are the photon energy, dispersion energy and oscillator energy, 

These parameters for each guest vapor are obtained by fitting Eq. (4) to the experimental RI data and are tabulated in Table 1. Using the dispersion relation in Eq. (4) and the constants in Table 1, the SPR response curves are simulated using an N-layer model for the multi-layer fiber tip structure. It should be noted that we adopt a 200 nm thick ZIF-8 layer for the simulations, in accordance with the experimental film thickness reported by Keppler et al.^21^ which forms the basis for our theoretical model.

**Table 1:** E_d_ and E_o_ values of each loaded combination extracted from the curve fitting.

Material	Fitted E_d_	Fitted E_o_
Empty ZIF-8	8.1663 ± 0.0173	10.1996 ± 0.0196
ZIF-8 + THF	7.3847 ± 0.0197	8.7887 ± 0.0206
ZIF-8 + DMF	9.5378 ± 0.0160	9.8273 ± 0.0149
ZIF-8 + Ethanol	9.9205 ± 0.0330	11.0690 ± 0.0337

### N-layer model

**Fig. 3:**
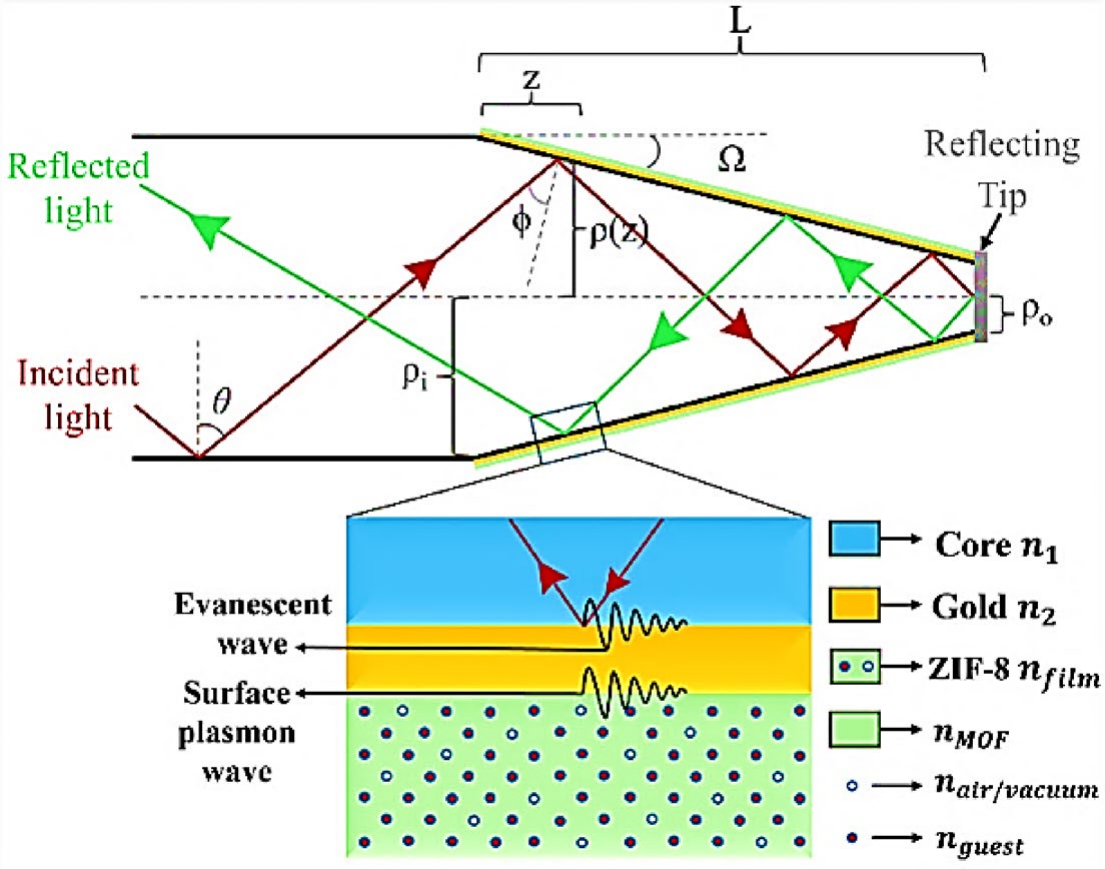
Schematic for the reflecting tip fiber optic probe with parameters.

To determine the reflection coefficient of the sensor, we employ a transfer matrix method based on an N-layer model, with the layers arranged along the z-axis. The transfer matrix method^7^, accounts for the interferences occurring in a thin film stack due to multiple reflections and transmissions, by solving Maxwell’s equations with boundary conditions, thereby yielding the overall reflectance curve for the multi-layer structure. The reflection of the proposed probe is calculated by the integral formula:

5 $$Tp = \frac{{\int_{0}^{L} {dz} \int_{{\phi _{1} \left( z \right)}}^{{\phi _{2} \left( z \right)}} {R_{p}^{{2N_{{ref}} }} } \frac{{n_{1}^{2} \sin \theta \cos \theta }}{{\left( {1 - n_{1}^{2} \cos ^{2} \theta } \right)^{2} }}d\theta }}{{\int_{0}^{L} {dz} \int_{{\phi _{1} \left( z \right)}}^{{\phi _{2} \left( z \right)}} {\frac{{n_{1}^{2} \sin \theta \cos \theta }}{{\left( {1 - n_{1}^{2} \cos ^{2} \theta } \right)^{2} }}d\theta } }}$$

where Tp is the normalized transmitted power, Rp is the Fresnel reflection coefficient for p‑polarized light, N_ref_ counts the number of reflections within the layer stack, n1 is the refractive index of the fiber core, and θ is the angle of incidence relative to the interface normal. The integration limits account for the angular power distribution of guided rays along the fiber length L. A more detailed derivation of the N‑layer formalism is provided in the Supplementary Information. All geometric and material parameters used in the simulation are schematically represented in Fig. 3. To calculate the SPR characteristics for the tapered optical fiber, we adopt the modelling framework developed by the group of Prof. Gupta^7^.

A TM polarized polychromatic light source is launched into the optical fiber using a microscopic objective, which focuses the beam onto the fiber core. The electromagnetic field subsequently propagates towards the tapered sensing region shown in Fig. 3. To accurately model the SPR response, the angular power distribution of the guided modes is considered, where each ray is characterized by a propagation angle θ before entering the taper. The taper geometry modifies these propagation angles, resulting in a distribution of effective incidence angles at the metal dielectric interface and thereby influencing the SPR excitation conditions. Further details of the angular distribution model are provided in the supplementary information.

## Results and discussion

**Fig. 4:**
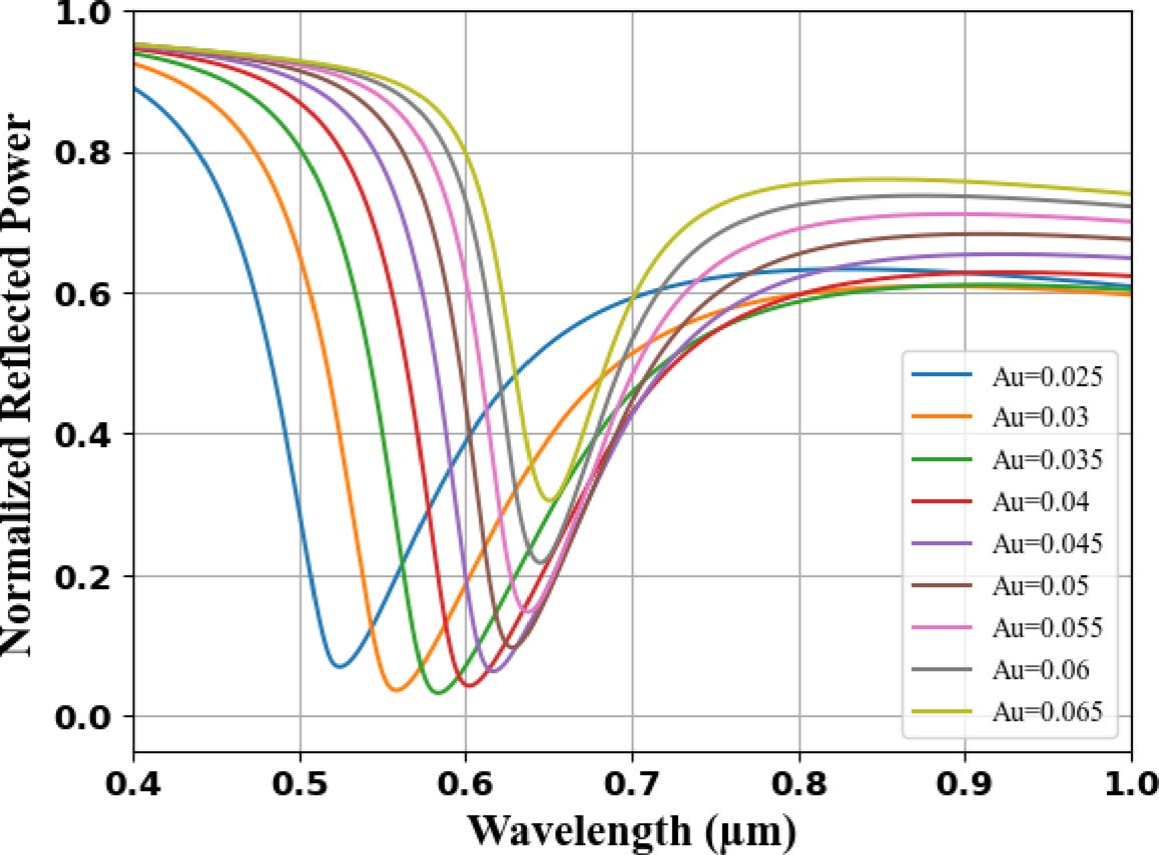
Optimization of Au layer thickness for the untapered SPR configuration with a core diameter of 600 μm, NA of 0.22, ZIF-8 of 200 nm thickness and the sensing region length of 1 cm.

In the simulation study, fiber optic SPR (FO-SPR) model was optimized and analyzed via wavelength interrogation using Python 3.9.13 (Python Software Foundation) within a Jupyter Notebook environment (version 3.4.4, Project Jupyter, supported by NumFOCUS), executed through the Anaconda IDE (conda 22.11.1, Anaconda, Inc.). Initially, SPR curves for the untapered fiber probe were simulated for a configuration with 600 μm core diameter, a 50 nm thick Au layer, a numerical aperture (NA) of 0.22 and a 1 cm long sensing regime. These parameters were selected to align with experimentally realizable conditions based on the availability of commercial optical fibers. Furthermore, the Au thickness was optimized while keeping the other geometrical parameters, such as fiber diameter and ZIF-8 thickness, constant. Figure 4 illustrates the optimization of the Au layer thickness, for the untampered SPR configuration, revealing that at 35 nm, the SPR dip exhibits the lowest reflected power.

**Fig. 5:**
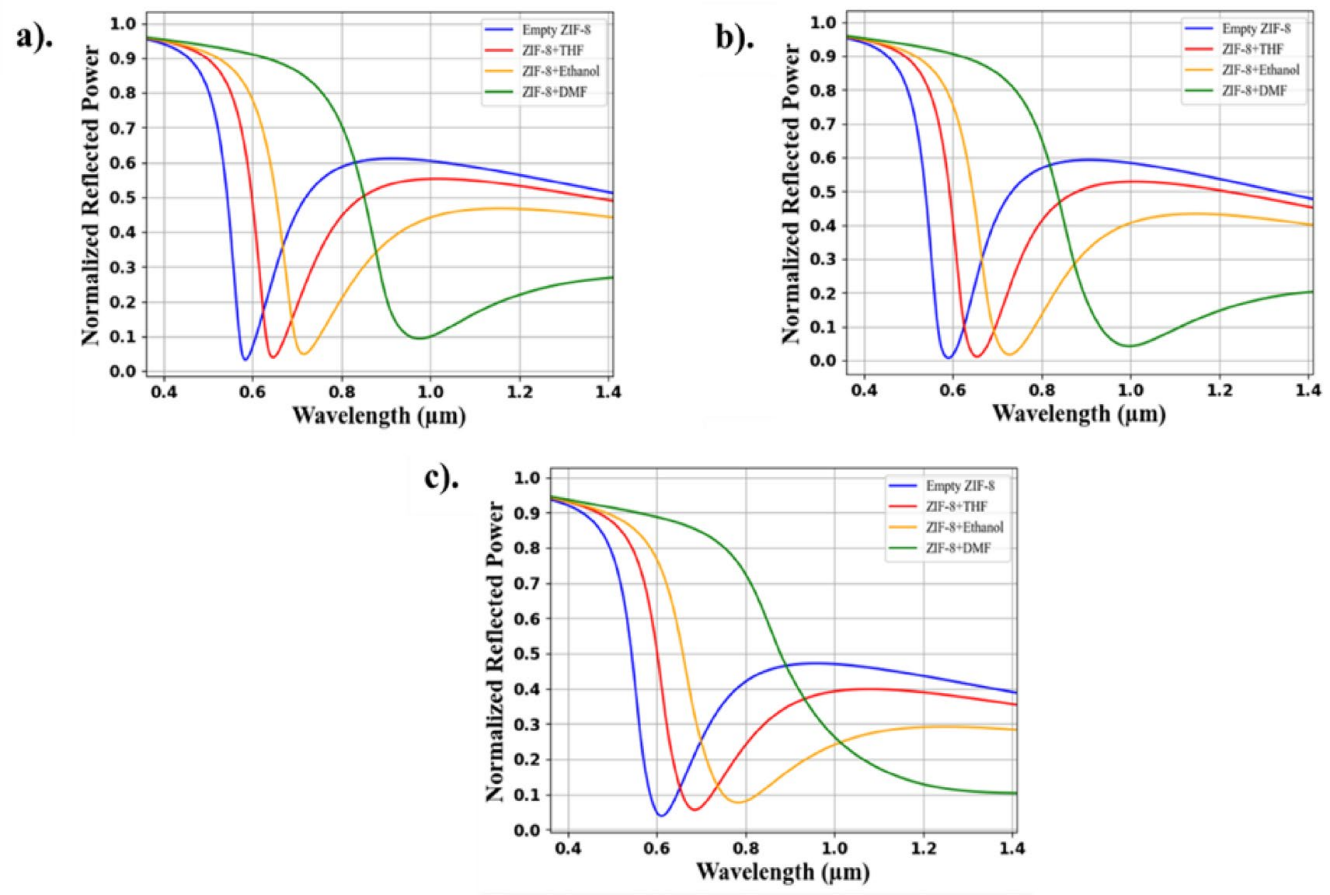
SPR plots of the tapered configuration with taper ratios (**a**) 1, (**b**) 1.5 and (**c**) 3.0.

The minimum reflected power (0.03246) is obtained at an Au layer thickness of 35 nm at a resonance wavelength of 0.5836 μm (583.6 nm). Any deviation from this optimal thickness, either by increasing or decreasing the Au layer thickness, results in higher reflectivity values and consequently weaker SPR excitation. For Au thickness below 35 nm, the metal film becomes insufficient to support efficient excitation and confinement of SPR, leading to a broader and less pronounced resonance dip. In contrast, for thickness greater than 35 nm, the penetration of the evanescent field through the metal layer is reduced, weakening the coupling between the guided optical mode and the surface plasmon wave. Therefore, an Au thickness of 35 nm provides the optimum balance between plasmonic confinement and field penetration, resulting in the deepest SPR resonance and strongest light plasmon coupling. Gold was selected as the plasmonic transduction layer owing to its favourable balance between sensitivity, chemical stability and long-term operational reliability. To evaluate the suitability of alternative plasmonic metals, additional simulations were performed using Ag and Cu under identical geometrical conditions, and the results are summarized in Table S2. The plots are mentioned in the supporting document (S5). Although Ag and Cu are attractive due to their lower cost and strong plasmonic response, Au exhibited the highest resonance wavelength sensitivity in the investigated tapered FO-SPR configuration. Using the optimized Au thickness, SPR spectra were then simulated for different ZIF-8-analyte combinations by varying the critical angle to locate the minimum reflectance (Fig. 5a). In the next stage, a tapered fiber probe structure was considered as shown in Fig. 1. The analysis was carried out as a comparison of three taper ratios: 1 (core diameter = 600 μm or untapered), 1.5 (core diameter = 400 μm), and 3 (core diameter = 200 μm). This comparison allows the evaluation of how core‑diameter reduction and increased evanescent field strength influence the SPR response, thereby providing a basis for sensitivity‑enhanced, analyte‑selective sensing.

It is worth noting that the taper ratio is defined as the ratio of the tapered core diameter to the initial core diameter. Figure 5a–c show the SPR spectra of the different taper profiles. The results indicate that increasing the taper ratio enhances sensitivity, however, it results in significant broadening of the SPR curves.

The number of reflections within the sensing region is inversely proportional to the local taper radius and can be expressed as:

$$\:{N}_{ref}\left(\theta\:,z\right)=\frac{L}{2\rho\:\left(z\right)\mathrm{tan}(\theta\:+{\Omega\:})}$$

where L is the sensing length, $\:\rho\:\left(z\right)$ is the local taper radius, $\:\theta\:$ is the incidence angle and $\:{\Omega\:}$ is the taper angle. Since increasing the taper ratio reduces the fiber radius, the number of internal reflections increases accordingly. The higher number of reflections enhances the interaction between the guided optical field and the plasmonic interface, improving sensitivity. However, SPR excitation occurs over a broader distribution of reflection angles and interaction lengths, producing overlapping resonance contributions. As a result, the SPR dip broadens with increasing taper ratio. Similarly, Bhatia et al., demonstrated sensitivity enhancement in a tapered fiber optic SPR sensor through the incorporation of a high-index dielectric layer, highlighting the role of dielectric engineering in improving plasmonic coupling and sensor performance^33^. To determine the operating conditions for each taper ratio, an angular sweep was first performed using the empty ZIF-8 configuration. It may also be noted that the incident angle corresponding to the deepest SPR resonance dip was selected as the operating angle for that particular taper ratio. This optimized angle was subsequently kept constant for all analyte simulations to ensure that the observed spectral variations originated solely from analyte-induced refractive index changes rather than changes in the excitation conditions. Using the optimized incident angle for each taper ratio, SPR spectra were subsequently calculated for different material combinations such as ZIF-8, ZIF-8 + THF, ZIF-8 + Ethanol, ZIF-8 + DMF, and the corresponding resonance wavelengths were obtained from the minimum reflected power values.

Consequently, obtaining a sharply defined SPR resonance becomes increasingly challenging for taper ratios exceeding 3, which establishes a practical upper limit for the design. To evaluate the trade-off between sensitivity enhancement and resonance broadening, the figure of merit (FOM) was calculated for each taper ratio. The FOM is defined as FOM = S / FWHM, where S is the sensitivity, and FWHM is the full width at half maximum of the SPR resonance dip. A higher FOM indicates better overall sensing performance by simultaneously accounting for resonance shift and spectral sharpness. The resonance wavelength and corresponding minimum reflectance power (Min Rp) values for each taper profile, for ZIF-8 and ZIF-8 loaded with ethanol, THF, and DMF, are summarized in Table 2.

**Table 2:** Results of the three tapered profile examined: summarising the resonance wavelength and minimum reflectance values.

Sensing Region	Taper ratio	λ_res_ (µm)	Min *R*_*P*_	Sensitivity (nm/RIU)	FWHM (nm)	FOM (RIU^−1^)
Empty ZIF-8	1	0.584	0.0325	3000	130.3	23.02
	1.5	0.590	0.0070	3100	134.8	22.99
	3	0.611	0.0386	3600	196.3	18.33
ZIF-8 + THF	1	0.646	0.0388	4000	161.3	24.79
	1.5	0.654	0.011	4200	173.2	24.24
	3	0.685	0.0560	5300	298.8	17.73
ZIF-8 + Ethanol	1	0.715	0.0490	6300	246.8	25.52
	1.5	0.727	0.0168	6600	267.9	24.63
	3	0.783	0.0766	9900	–	–
ZIF-8 + DMF	1	0.975	0.0935	–	–	–
	1.5	0.997	0.0422	–	–	–
	3	–	–	–	–	–

**Fig. 6:**
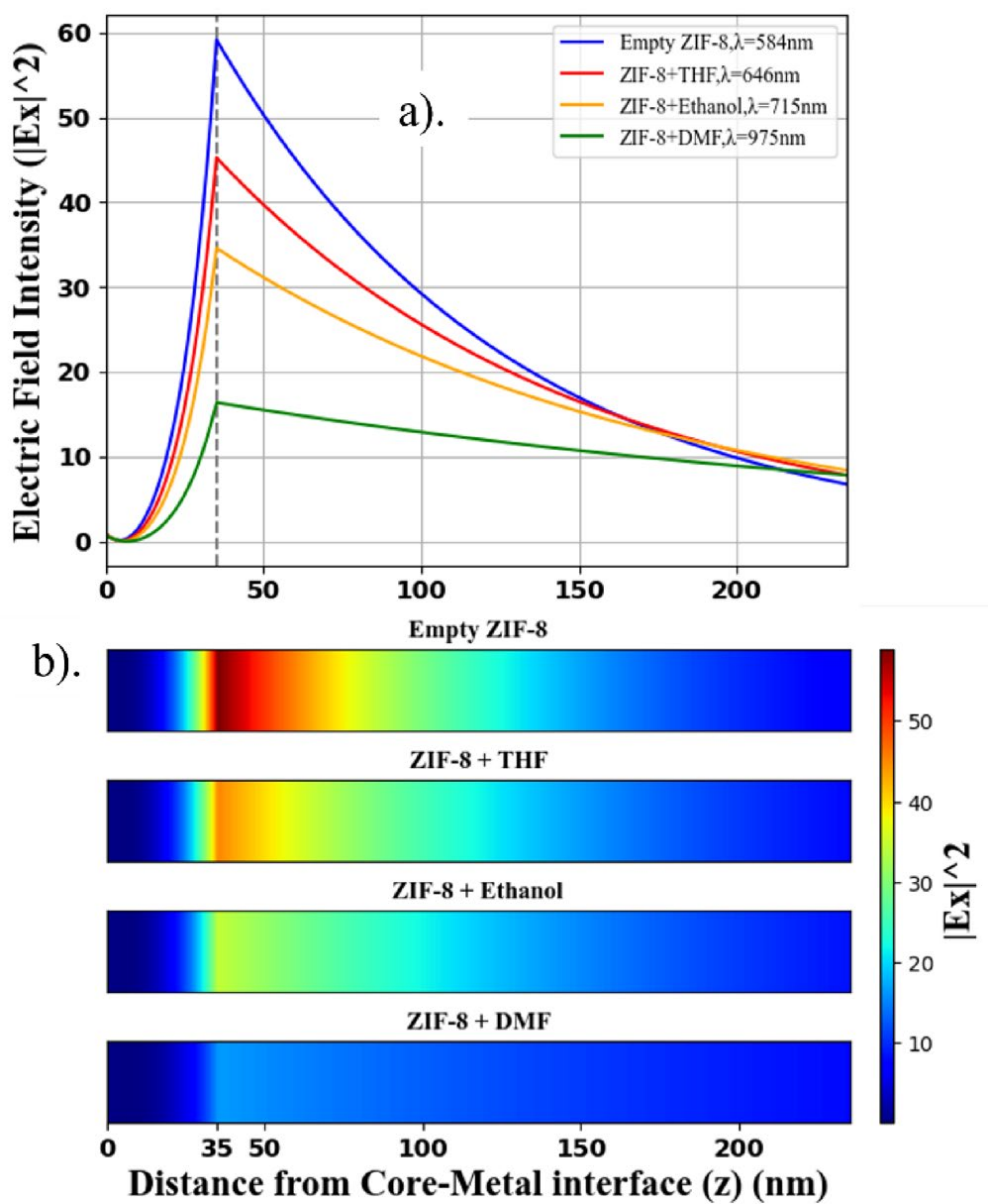
(**a**) Electric field distribution of the proposed probe with empty ZIF-8 and after the interaction of vapors (**b**) colormap profile of electric field distribution.

As evident from Fig. 5; Table 2, all analyte-loaded ZIF-8 configurations exhibit a red shift in the resonance wavelength relative to the empty ZIF-8 layer, also the values mentioned in Table S1. This behaviour originates from the pore-filling mechanism of the ZIF-8 sensing layer, where the adsorption of guest molecules within the porous framework replaces the vacuum-like cavities and increases the effective refractive index of the MOF film. The resulting refractive index modulation alters the SPR phase-matching condition, causing the resonance wavelength to shift towards longer wavelengths. Furthermore, the magnitude of the red shift depends on the effective refractive index of the analyte-loaded ZIF-8 layer. As shown in Fig. 2, DMF induces the largest refractive index increase in the ZIF-8 layer, followed by ethanol and THF^21^. Consequently, DMF produces the largest resonance wavelength shift among the investigated analytes, as observed in Fig. 5; Table 2. The broad SPR spectrum observed for the ZIF-8 + DMF configuration is attributed to the relatively high effective refractive index of the DMF-loaded ZIF-8 layer. As the refractive index of the sensing layer approaches that of the fiber core, the refractive index contrast at the sensing interface decreases, weakening the sharpness of the SPR coupling condition. Consequently, the resonance condition becomes less well defined and SPR excitation occurs over a broader wavelength range, leading to an increased resonance linewidth and a broader SPR dip. Therefore, although DMF exhibits the largest resonance wavelength shift, it also produces a broader resonance spectrum and a lower figure of merit compared with THF and ethanol. In addition to sensitivity enhancement, the resonance bandwidth was evaluated through the FWHM of the SPR dip. Although increasing the taper ratio results in larger resonance wavelength shifts, the SPR curves become progressively broader, as shown in Fig. 5. Therefore, the FOM does not necessarily increase proportionally with the taper ratio. The calculated FOM values indicate the optimum balance between sensitivity and spectral resolution for the investigated taper configurations. To further understand the origin of the observed SPR wavelength shifts, the electric field distribution of the proposed sensor was analyzed. For the untapered (or straight) profile, the electric field intensity as a function of distance $\:z$ from the fiber core-metal interface was computed numerically in Fig. 6 using the algorithm proposed by Shalabney and Abdulhalim^34^, based on Abele’s transfer matrix method^7,28^ for wave propagation in multilayer structures. The electric field distribution confirms that for all combinations, the electric field intensity peaks at the metal-sensing layer interface, located 35 nm from the fiber core. At this interface, the evanescent field penetrates the ZIF-8 sensing layer and interacts with the guest molecule adsorbed within its pores. Upon analyte adsorption, the effective refractive index of the ZIF-8 layer increases due to the replacement of vacuum like pore volume by guest molecules. This modifies the dielectric environment adjacent to the Au surface and consequently alters the propagation constant of the surface plasmon wave. As a result, the electric field distribution at the Au/ZIF-8 interface is altered, leading to changes in field confinement and penetration into the sensing layer. Since SPR excitation occurs when momentum matching is satisfied, the modified dielectric environment shifts the resonance condition towards longer wavelengths, resulting in the observed red shift. The stronger refractive index perturbation produced by analyte loading, therefore, manifests as both a redistribution of the electric field intensity and a corresponding SPR wavelength shift.

To quantitatively evaluate the sensing performance of the proposed sensor, sensitivity was calculated using two complementary approaches. In the first approach, the sensitivity was determined from the resonance wavelength variation associated with changes in the guest molecule volume fill factor within the ZIF-8 layer. In the second approach, the sensitivity was calculated by varying the refractive index of the sensing layer by Δn = 0.01 and evaluating the corresponding resonance wavelength shift. The calculated sensitivity values are summarized in Table S3 in the supporting information.

### Effect of ZIF-8 fill factor on SPR sensing

The sensitivity of an SPR sensor is calculated as the ratio of change in resonant wavelength to the corresponding change in RI of the sensing layer, S = Δλ/ΔRI. In the present configuration for chemical vapor detection, the RI of ZIF-8 varies with the volume of the guest molecules (gas or vapor) loaded into its pores, effectively changing the fill factor of the MOF film. The influence of this fill factor on the change in resonance wavelength was therefore investigated, initially using THF as the target vapor.

6 $$\:{f}_{ZIF-8}=\frac{{RI}_{film}^{2}-{RI}_{guest}^{2}}{{RI}_{ZIF-8}^{2}-{RI}_{guest}^{2}}$$

**Fig. 7:**
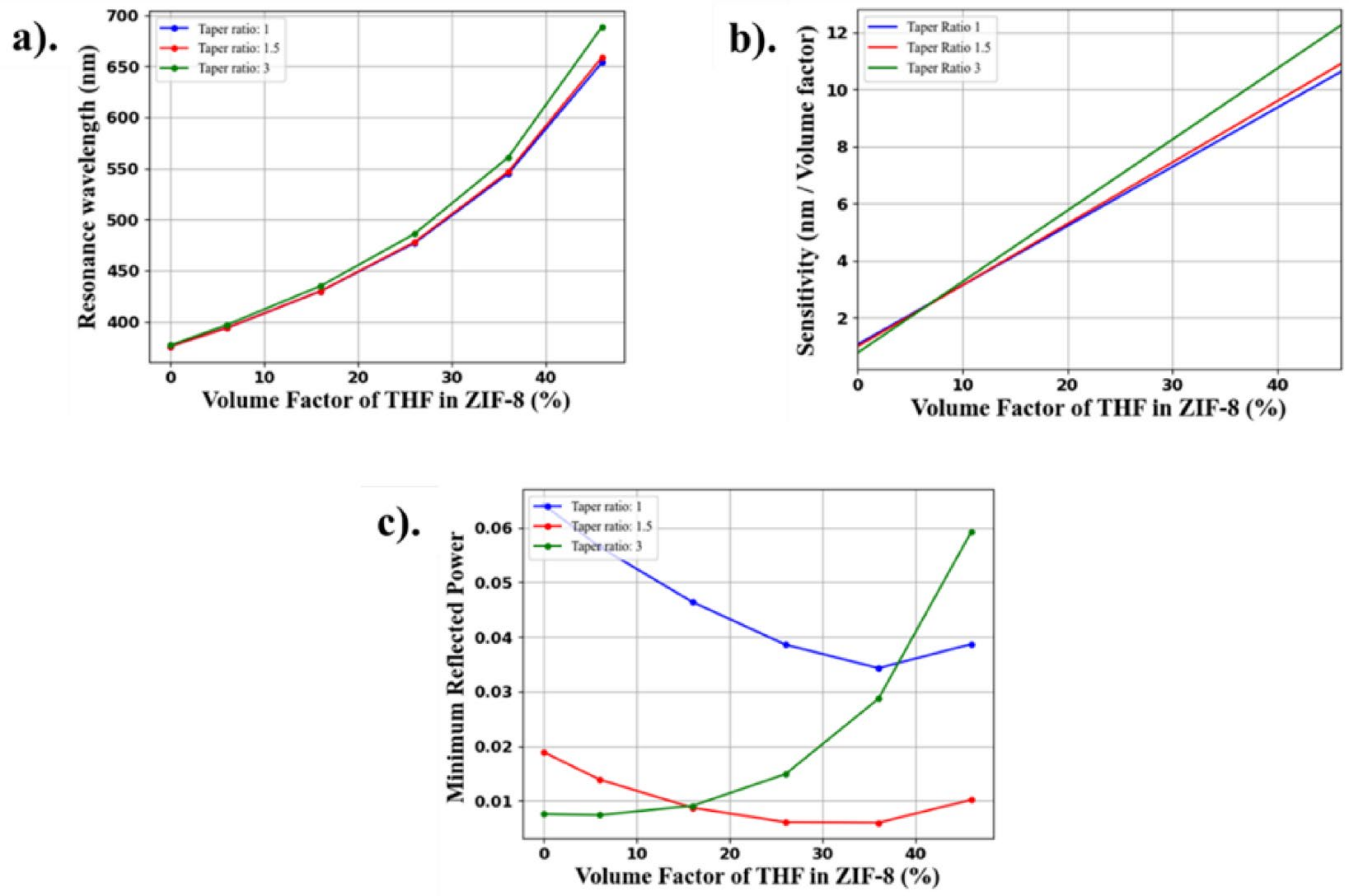
(**a**) Resonance wavelength, (**b**) sensitivity and (**c**) minimum reflected power for three different taper ratios in the SPR configuration with THF guest chemical having various volume factors in ZIF-8.

The volume factors of the ZIF-8 sensor layer within the configuration is expressed as:

For THF, the volume factor of guest in the ZIF-8 layer is then calculated as, *f*_*Guest*_ *= *1 -* f*_*ZIF−8*_, where *RI*_*ZIF−8*_ = 1.355, *RI*_*THF*_ = 1.40 for λ = 589 nm)^2^, and *RI*_*film(ZIF−8+THF)*_ *=* 1.376. Using the formulae, we calculated the values as *f*_*ZIF−8*_ ≈ 0.5373 and *f*_*THF* ≈_ 0.4626. This corresponds to the THF fill fraction of approximately 46.26% within the ZIF-8 layer, assuming complete pore filling with no additional air gaps. Figure 7a shows the effect of varying the THF fill factor on the SPR response in terms of resonance wavelength and minimum reflected power for three different taper ratios. The complete set of SPR curves for THF at different volume fractions and taper ratios is provided in the supporting information (Figures S1–S4). To consistently describe the dispersive behaviour of the ZIF‑8 and guest film across all fill factors, Eq. (3) is extended into a volumetric form that incorporates the single oscillator model. This yields the modified equation for analysis, and here, the volume fraction of THF is further varied from 0 to 46.26% with the resulting SPR curves simulated for different taper ratios.

7 $$\:{n}_{film}=\sqrt{{f}_{MOF}\left[\frac{{E}_{d}{E}_{o}}{{E}_{o}{\left[\frac{hc}{\lambda\:}\right]}^{2}}+1\right]+{f}_{guest}{{n}_{guest}}^{2}+(1-{f}_{MOF}-{f}_{guest}){{n}_{vacc}}^{2}}$$

Figure 7 illustrates that as the taper ratio increases, the change in resonant wavelength also increases, leading to higher sensitivity of the sensor. However, at higher taper ratios, the SPR curve broadens, which degrades the RI resolution of the sensor. Specifically, as the taper ratio increases from 1 to 1.5, the minimum reflected power approaches zero with increasing fill factor; indicating stronger SPR excitation and deeper penetration of the plasmonic field into the ZIF‑8 layer. However, beyond a taper ratio of 1.5 up to 3, the minimum power begins to shift away from zero, implying a trade‑off between sensitivity and curve contrast. Consequently, an optimal taper ratio must be selected for each specific chemical vapor analyte to balance high sensitivity against acceptable RI resolution.

Figure 7b represents the calculated sensitivity of the sensor probe as a function of THF volume fraction, demonstrating a monotonic increase with fill factor. To verify the generality of this behaviour, an additional fill factor analysis was performed for DMF and the corresponding sensitivity values are provided in the supporting information Table (S1 and S3) and figure S6. Keppler et al. experimentally determined the RI of ZIF-8 thin films before and after loading with several vapors including toluene, phorbol 12-myristate 13-acetate, iodobenzene and dimethyl sulfoxide, apart from THF, ethanol, and DMF. It has been found that the effective index of ZIF-8 can be tuned from 1.355 to 1.421 in order for the material to support the SPR mechanism, as the limit lies within the RI value of fiber core. This explains the increasingly broadened SPR curves observed at higher RI values. It is noteworthy that the minimum reflected power increases at higher THF volume fractions, particularly for a taper ratio of 3. This behaviour is associated with the progressive increase in the effective refractive index of the analyte-loaded ZIF-8 layer as the pore filling factor increases. This results in a broadening of the SPR resonance, as evident from the spectra presented in Figure S4 of the supporting information. The resonance broadening causes the SPR dip to become less pronounced, leading to an increase in the minimum reflected power. Therefore, although increasing the THF fill factor continues to shift the resonance wavelength and enhance sensitivity, the SPR coupling becomes less sharply defined at higher fill factors, resulting in a broader resonance profile and a shallower reflectance minimum. Once the effective index of the sensing layer approaches or exceeds the core index, the evanescent‑field confinement weakens, and the SPR dip broadens markedly. Therefore, any chemical vapor analytes that alter the ZIF-8 RI beyond this operational window thus fall outside the detectable range of the proposed sensing platform. It must be noted that this study did not employ density functional theory (DFT) or molecular-scale simulations to model the adsorption or desorption kinetics of the target vapor. Instead, we modelled the pore-filling effect and its impact on the SPR response, relying on deep experimental characterization and data reported on the refractive index variations of ZIF-8 upon guest molecule loading^21^, focusing on how such changes manifest in the SPR signal. Within this modelling framework, the analysis of the refractive index changes induced by individual guest molecules in the ZIF-8 layer and their corresponding influence on the SPR response. The current study does not address mixed vapor environments. Consequently, the observed differences in resonance wavelength among THF, ethanol, and DMF should not be interpreted as evidence of intrinsic chemical selectivity, but rather as a distinct SPR response resulting from analyte-induced index modulation of the ZIF-8 layer. Furthermore, the adsorption-induced refractive index data employed in this study are derived from single vapor measurements reported by Keppler et al.^21^, The mixed vapor adsorption is not considered. In practical sensing scenarios, discrimination between vapors that produce similar response shifts may require additional information, such as adsorption kinetics, sensor arrays employing different MOFs or taper geometries, or advanced pattern recognition and machine learning approaches. To assess the performance of the proposed sensor relative to existing vapor sensing platforms, a comparison with representative vapor sensors reported in the literature is presented in Table 3.

**Table 3:** Comparison between the proposed work with existing literatures

Working principle	Configuration	Transducing unit	Analyte/target vapor	Sensing range	Sensitivity	Reference
SPR	D-shaped fiber optic SPR sensor incorporating double perovskite halide (DPH) biolayers, Cs_2_AgBiY_6_ (Y = Cl, Br, I)	D-shaped optic fiber, Ag, Ag-Cs_2_AgBiCl_6_, Ag-Cs_2_AgBiBr_6_, and Ag-Cs_2_AgBiI_6_	Breast cancer and adrenal gland biomarkers	–	Ag - 6001 nm/RIU, Ag-Cs_2_ Ag Bi Cl_6_ - 6512 nm/RIU, Ag-Cs_2_ Ag Bi Br_6_ - 6930 nm/RIU, Ag-Cs_2_ Ag Bi I_6_ - 7898 nm/RIU	^35^
SPR	D-shaped, grating and non-grating palladium (Pd) over Ag coated fiber SPR sensor.	30 nm Ag layer, 100 nm residual cladding, 30 nm air gap width and 20 Pd gratings	Carbon monoxide (CO)	LOD - 0.82 ppm CO	non-grating - 6300 nm/RIU after grating - 7100 nm/RIU	^36^
SPR	Back-reflecting fiber optic SPR sensor with Au coated ZIF-8 framework.	Au, ZIF-8 framework	Ethanol, 1-propanol, or 1-butanol liquid solvent Heterogeneous and Homogeneous Samples.	RI = 1.3727−1.4596 nm	–	^6^
Intensity-modulation of circular interference fringe pattern	Intensity-modulated tapered-tip optical fiber refractometer	Back-reflecting tapered-tip optical fiber.	salt-water (NaCl) solutions	n = 1.33102 to 1.33114 RIU with steps of 2 × 10^−5^ RIU.	24,160 dB/RIU	^10^
SPR	SPR based reflective tapered fiber optic sensor with Au and ZIF-8 framework	Au, ZIF-8 framework	THF, DMF and Ethanol vapours	n = 1.355 to 1.421 RIU	THF - 5003 nm/RIU, Ethanol- 9900 nm/RIU, DMF - 5.698 nm/Volume factor	This work

It is also worth noting that MOFs have attracted considerable attention for vapor sensing because of their high porosity, tunable host–guest interactions, and strong adsorption characteristics. At the same time, sensor performance depends strongly on the fiber taper geometry and the interaction between the guided light and the MOF layer. Prior works have addressed components of this problem in parts: tapered FO-SPR sensing frameworks^34^, refractive index modulation in ZIF-8 during vapor adsorption^21^, MOF adsorption-dependent RI calculation methods^20^, and ZIF-8-integrated SPR/Fabry-Perot sensors for VOC detection^16,17^. However, a systematic theoretical analysis combining a reflective tapered FO-SPR configuration with experimentally determined refractive index changes of ZIF-8 remains limited. In this work, we integrate experimentally measured ZIF-8 refractive index changes into a reflective tapered FO-SPR model to study how taper ratio affects resonance wavelength, sensitivity, spectral broadening, and figure of merit for different vapor analytes. The study provides a unified theoretical framework that links MOF adsorption behaviour to tapered FO-SPR performance and offers design guidance for future experimental realization.

## Conclusion

The study presented a theoretical evaluation of an SPR-based refractometric sensor that integrates a tapered optical fiber configuration with a reflective tip and a gold-MOF sensing layer for chemical vapor detection. The SPR response of the sensor was systematically analyzed by varying both the taper ratios of the fibre and the volume fractions (fill factor) of chemical analyte in the ZIF-8 layer in the proposed configuration. Results revealed that increasing the taper ratio enhances the resonance wavelength shift, significantly improving sensor sensitivity. At the same time, fill-factor analysis confirms the critical role of ZIF-8 porosity in modulating the effective refractive index and, consequently, the SPR response. The results demonstrate a clear correlation between fiber tapering, pore-filling effects in ZIF-8, and sensor sensitivity, validating the importance of the present study and expanding the scope and applicability of SPR-based fiber optic refractometric sensors. Optimizing the taper ratio and exploiting the pore-filling effect of ZIF-8 leads to significant resonance wavelength shifts and higher sensor sensitivity across the visible spectral range. The proposed sensor could enable compact, portable, and non-invasive real-time vapor monitoring, offering a lightweight alternative to conventional electrochemical sensors, with lower power. Future work can focus on further optimization of the taper parameters, exploration of alternative MOFs with diverse porosity and host-Guest interaction to enhance sensitivity and selectivity, along with direct experimental demonstration of the tapered platform. It should be noted that the present study is primarily based on theoretical modelling and numerical simulations of a reflective tapered FO-SPR sensor functionalized with a ZIF-8 sensing layer. Therefore, experimental validation through fabrication and characterization of the proposed sensor is necessary to verify the predicted performance under practical operating conditions. In addition, the current analysis is limited to the specific reflective fiber tip configuration and single vapour adsorption scenarios considered in this work. Factors such as mixed vapor interference, fabrication tolerances, non-uniform MOF deposition, and long-term operational stability will be considered in future work. The present work is primarily based on theoretical modelling and numerical simulations of a reflective tapered FO-SPR sensor functionalized with a ZIF-8 sensing layer. While the proof-of-concept experiment demonstrates the feasibility of the sensing concept, comprehensive experimental validation of the optimized tapered probe configuration remains to be performed, as detailed in the supplementary information. Furthermore, the current analysis is limited to single vapor adsorption scenarios and does not consider mixed vapor interference, concentration dependent measurements, limit of detection analysis, fabrication tolerances, and long-term stability effects. Extending this approach to real-time sensing in complex environments, combined with machine learning-based data analysis and calibration, could significantly further advance the sensor’s precision, selectivity, and practical applicability in environmental monitoring.

## Supplementary Information

Below is the link to the electronic supplementary material.

Supplementary Material 1

## Data Availability

All data generated or analyzed during this study are included in this published article and its supplementary information file.
