# Supplementary material for: Metal organic framework functionalized reflective tapered fibre-optic surface plasmon resonance based refractometer for vapor sensing
Source: Sci Rep. 2026 Jul 7;16:21012. doi: 10.1038/s41598-026-61422-9 (PMC13342587; doi:10.1038/s41598-026-61422-9)
Supplement: Supplementary file 1 — Supplementary Material 1 [file 41598_2026_61422_MOESM1_ESM.docx]

**Support data:**

**S1. N – Layer Model:**

To understand the N- layer model, let us consider a random layer (say jth) between the stack of N-layers having the constants such as thickness, RI, permeability, and permittivity as dj, nj, μj, and εj, respectively. Further, the tangential components of the electric and magnetic fields at the first boundary E1 and H1 respectively, are related with the corresponding values at the final boundary, EN−1 and HN−1 as follows:

| $\left( \frac{E_{1}}{H_{1}} \right)=M\left( \frac{E_{N-1}}{H_{N-1}} \right)$ | (5) |
| --- | --- |

where M is the abeles matrix or the transfer matrix with a combinational structure for the transverse electric (TE) and transverse magnetic (TM) modes as:

| $M=\prod_{j=2}^{N-1} M_{j}=\prod_{j=2}^{N-1} \left[ \begin{matrix} \cos\beta_{j} & \frac{-i\sin\beta_{j}}{q_{j}} \\ -i q_{j}\sin\beta_{j} & \cos\beta_{j} \end{matrix} \right]$ | (6) |
| --- | --- |
|  |  |

where, the propagation constant for each interference layer is given below:

| $\beta_{j}=\frac{2\pi d_{j}}{\lambda} \sqrt{\varepsilon_{j}-n_{1}^{2}{sin}^{2}\theta_{1}}$ | (7) |
| --- | --- |

and $\theta_{1}$ is the angle of the ray from normal at the first interference. Further, in the case of a TM-polarized light (which is essential for SPR realization), the amplitude of reflection coefficient *rp,* is given by,

| $rp=\frac{\left( M_{11}+M_{12}q_{N} \right)q_{1}-\left( M_{21}+M_{22}q_{N} \right)}{\left( M_{11}+M_{12}q_{N} \right)q_{1}+\left( M_{21}+M_{22}q_{N} \right)}$ | (8) |
| --- | --- |

were,

| $q_{j}=\sqrt{\frac{\mu_{j}}{\varepsilon_{j}}}\cos\theta_{j}=\frac{\sqrt{\varepsilon_{j}-n_{1}^{2}{sin}^{2}\theta_{1}}}{\varepsilon_{j}}$ | (9) |
| --- | --- |

Hence, the reflectance for p-polarized light is evaluated as:

| $R_{p}=\left\vert r_{p} \right\vert^{2}$ | (10) |
| --- | --- |

The angular power distribution of rays guided in the fiber with $\theta$ as the angle of the ray before it enters the tapered part,

| $dP\propto\frac{n_{1}^{2}\sin\theta\cos\theta}{\left( 1-n_{1}^{2}{cos}^{2}\theta\right)^{2}}d\theta$ | (11) |
| --- | --- |

Assuming adiabatic tapering, the taper radius at a distance z from the input end of the taper region is denoted as ρ(z), and the angle of the ray in the tapered region will be transformed to,

| $ф\left( z \right)={cos}^{-1} \left[ \frac{\rho_{i}\cos\theta}{\rho\left( z \right)} \right]-{tan}^{-1} \left[ \frac{\rho_{i}-\rho_{o}}{L} \right]$ | (12) |
| --- | --- |
|  |  |
|  |  |

where $\rho_{i}$ and $\rho_{o}$ are the radii of the taper input (z = 0) and taper output (z = L) regime, and L is the sensing regime length or the taper length. We are considering a linear taper profile, characterized by a varying taper radius as:

| $\rho\left( z \right)= \rho_{i}-\frac{z}{L}\left( \rho_{i}-\rho_{o} \right)$ | (13) |
| --- | --- |

The range of incident angles of the ray entering the input end of the taper ($\theta_{1}, \theta_{2})$ will become [$\phi_{1}\left( z \right), \phi_{2}\left( z \right)]$ at a distance $z$ in the tapered region due to the variations in core radius in the tapered regime. Since the probe has a reflective end the incident ray will reflect back into the core and then to the opening end of the fiber, where a spectrometer will measure the transmitted power at each wavelength as:

| $Tp=\frac{\int_{0}^{L} dz\int_{\phi_{1}\left( z \right)}^{\phi_{2}\left( z \right)} R_{p}^{2N_{ref}}\frac{n_{1}^{2}\sin\theta\cos\theta}{\left( 1-n_{1}^{2}{cos}^{2}\theta\right)^{2}}d\theta}{\int_{0}^{L} dz\int_{\phi_{1}\left( z \right)}^{\phi_{2}\left( z \right)} \frac{n_{1}^{2}\sin\theta\cos\theta}{\left( 1-n_{1}^{2}{cos}^{2}\theta\right)^{2}}d\theta}$ | (14) |
| --- | --- |
|  |  |

where ф_1_(z), ф_2_(z) can be obtained by substituting the value of $\theta$ in the equation (12) keeping limits like for ф_1_(z) for incident angle and ф_2_(z) for$\theta= \frac{\pi}{2}$. The number of reflections the incident ray at an angle $\theta$ undergoes in the sensing regime with length L and radius $\rho\left( z \right)$ is given by,

| $N_{ref}\left( \theta,z \right)=\frac{L}{2\rho\left( z \right)\tan\left( \theta+\Omega\right)}$ | (15) |
| --- | --- |

and due to the reflecting tip $N_{ref}$ is doubled. Here Ω is the taper angle, which is given by

| $\Omega={tan}^{-1} \left[ \frac{\rho_{i}-\rho_{o}}{L} \right]$ | (16) |
| --- | --- |

**S2. Results and Discussion**

**S3. Proof-of-concept Experiment**

The experimental investigation was conducted using an untapered fiber tip SPR probe. The objective of this proof-of-concept study was to verify the feasibility of the Au/ZIF-8 sensing architecture and the pore-filling-induced SPR modulation mechanism predicted by the theoretical model. To prepare the sensor probes, optical fibers were first sectioned into 10 cm segments, followed by the mechanical stripping of 1 cm of cladding from the distal ends to expose the silica core. The stripped segments were then submerged in a freshly prepared acidic Piranha solution for 1 hour at room temperature to achieve thorough surface cleaning and hydroxylation. Following the etching process, the fibers were rinsed extensively with deionized water and subsequently dehydrated in absolute ethanol to prepare the surface for further material deposition. Gold coating was carried out by two steps of physisorption: seeding followed by growth. For the seeding solution, 12.5 µL of 1 mM NaBH₄ was mixed with 2.5 µL of gold chloride and 485 µL of ultrapure water. The growth solution was formulated using the same volume ratios, with hydrogen peroxide replacing NaBH₄. The fiber end was first immersed in the growth solution for 1 minute and then in the seed solution, followed by thorough rinsing drying at 80 °C in vacuum oven for 30 minutes. ZIF-8 was synthesised and deposited on the gold-coated optical fibre via an in-situ growth method. A solution of zinc nitrate hexahydrate (59.498 mg) in 5 mL of deionized water was prepared, alongside a separate solution of 2-methylimidazole (65.6 mg) in 5 mL of deionized water, corresponding to metal-to-linker molar ratio of 1:16. The two solutions were mixed under ambient conditions, and the gold-coated optical fiber was immediately immersed into the resulting solution to enable in-situ nucleation and growth of ZIF-8 on the gold surface. Figure S7(a) shows the scanning electron microscopy (SEM) micrograph of the resulting ZIF-8 structure grown over Au (~ 30 nm) immobilized fiber tip surface for sensing. The image reveals the porous aggregated ZIF-8 structures that can enhance vapor sensing performance by increasing accessible surface area, improving diffusion pathways, and supporting stronger plasmonic interactions.

The prepared sensor probe was subsequently employed for ethanol and tetrahydrofuran (THF) sensing, where the sensing response arises from analyte adsorption within the porous ZIF-8 framework. Figure S7(b) displays the normalized reflectance spectra corresponding to ZIF-8/Au/tip probe at different fabrication steps and sensing stages. The initial Au growth phase (black curve) shows a characteristic reflectance minimum at 535.62 nm. Upon successful integration of ZIF-8 (red curve), a significant red shift of 31.89 nm to 567.51 nm is observed, which is attributed to the increased optical thickness and RI of the composite Au-MOF structure. The broadening of the reflectance dip and the intensity changes for the ZIF-8 curve indicate an increase in surface roughness or slight structural disorder typically associated with the nucleation of MOF crystals. Exposure of the ZIF‑8‑functionalized probe to THF and ethanol vapors produced additional red shifts in the resonance wavelength, confirming the feasibility of refractometric vapor sensing with this platform. It should be noted that these experiments were performed using an untapered probe configuration and therefore do not directly validate the sensitivity enhancement predicted for the tapered geometry. Instead, the measurements establish the feasibility of the ZIF-8 functionalized SPR sensing mechanism and provide a baseline for future implementation of the reflective tapered FO-SPR platform proposed in this work. Furthermore, the present proof-of-concept experiment does not include concentration-dependent calibration measurements, limit-of-detection estimation, or mixed-vapour interference studies. Consequently, the experimental results should be interpreted as a demonstration of the sensing mechanism rather than a complete evaluation of practical sensing performance. These preliminary results validate the theoretical model’s prediction that guest‑molecule adsorption and pore filling within ZIF‑8 modulate the SPR response. In the future, we plan to extend these experimental measurements to the proposed reflective‑tapered tip FO‑SPR geometry to directly benchmark the theoretical sensitivity and selectivity predictions.

**
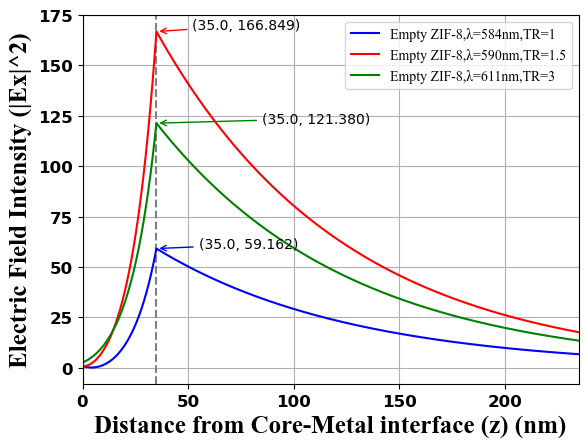
S2**. **Results and Discussion: Diagrams**


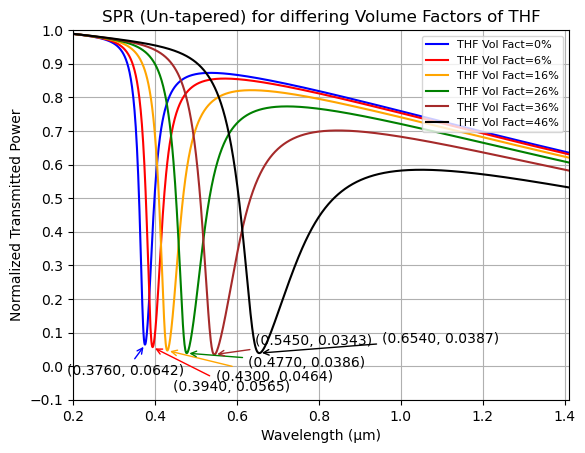
Figure S1. E-Field distribution of empty Zif-8 film for different Taper ratios

Figure S2. SPR plots for different Fill factors, D=600μm, Incident angle:**82.5°**


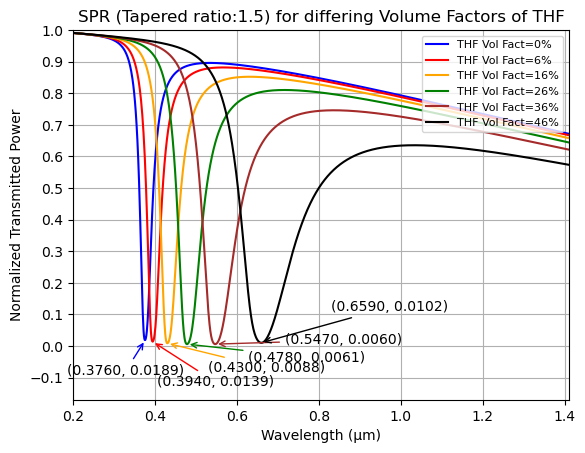


S3. SPR plots for different Fill factors, ρ_o_=200μm, Incident angle:**86.4°**


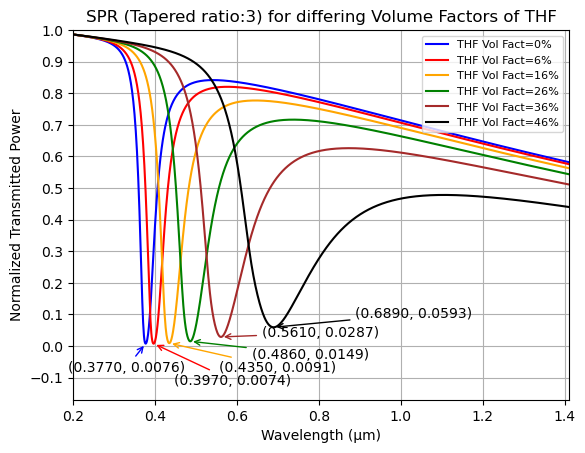


S4. SPR plots for different Fill factors, ρ_o_=100μm, Incident angle:**87.3°**


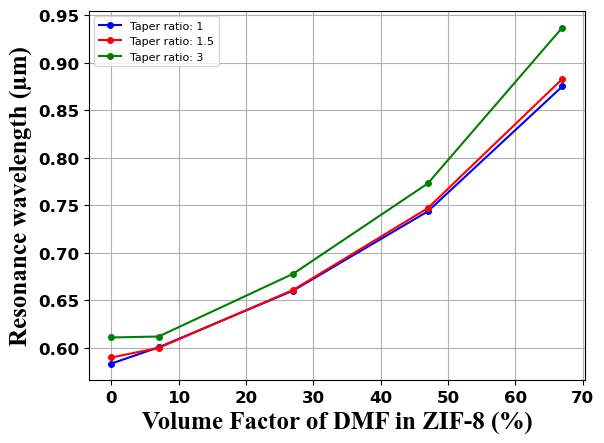


**(a)**


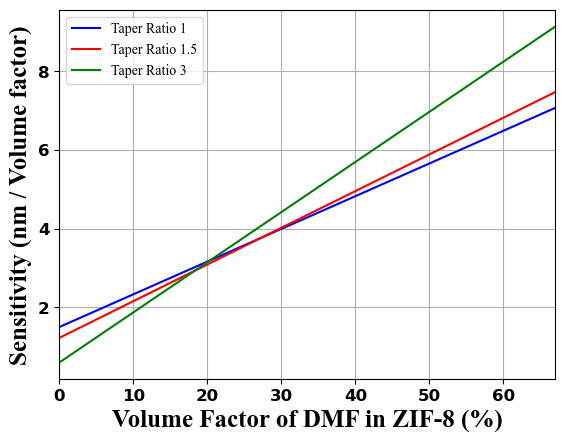


**(b)**


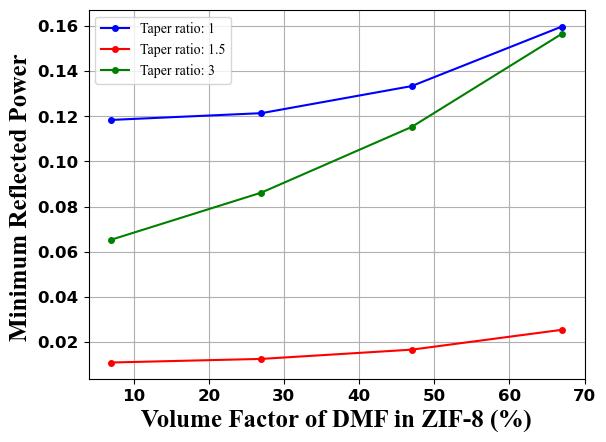


**(c)**

S5. (a) Resonance wavelength, (b) sensitivity and (c) minimum reflected power for three different taper ratios in the SPR configuration with DMF guest chemical having various volume factors in ZIF-8.

(b)


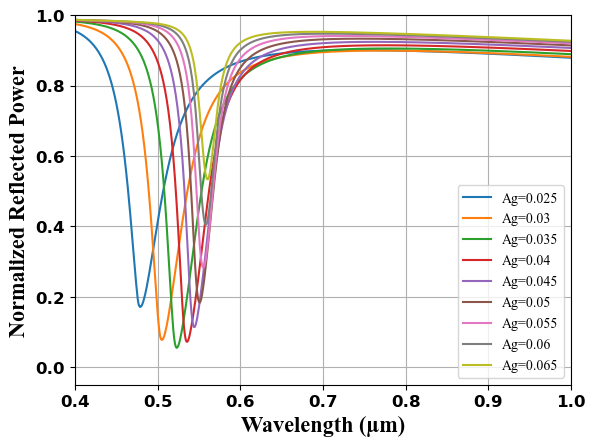


(a)


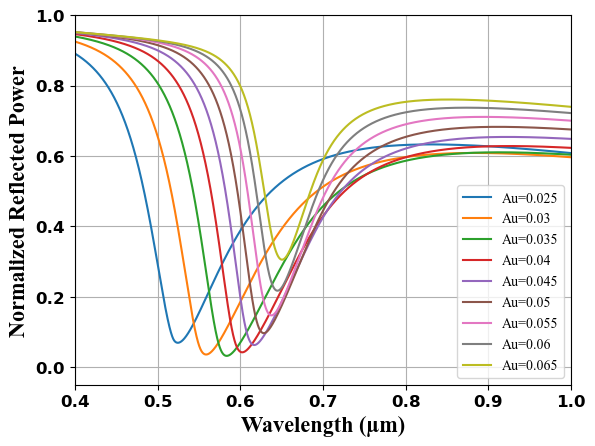

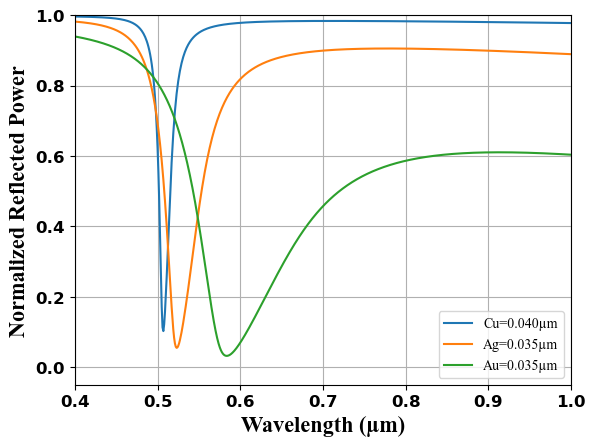


(d)


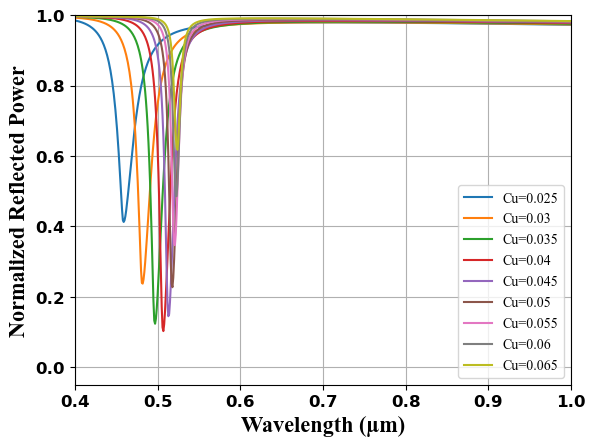


(c)


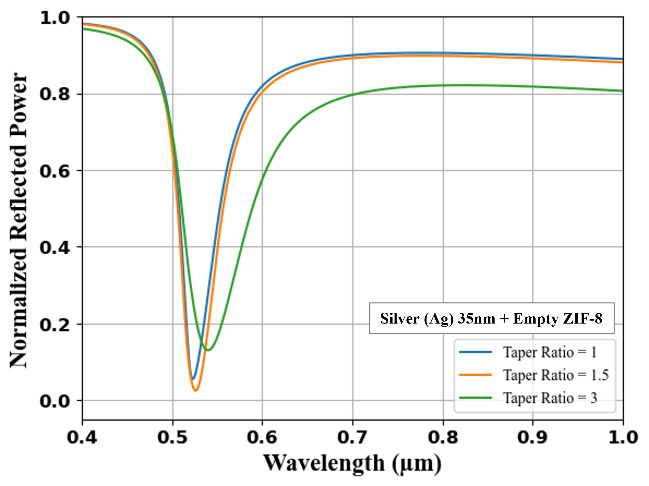


(f)


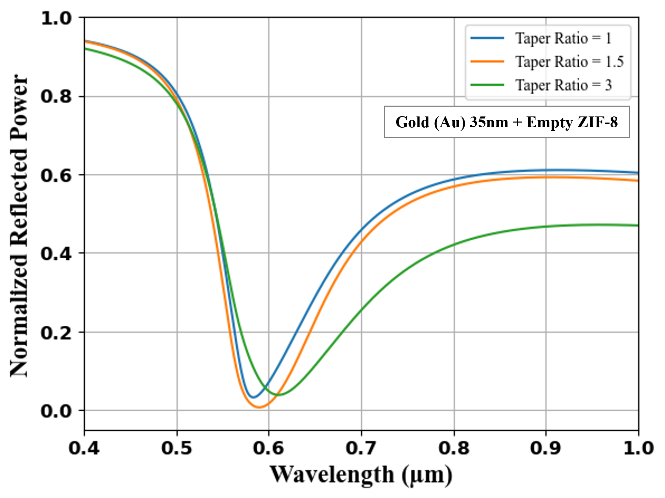


(e)


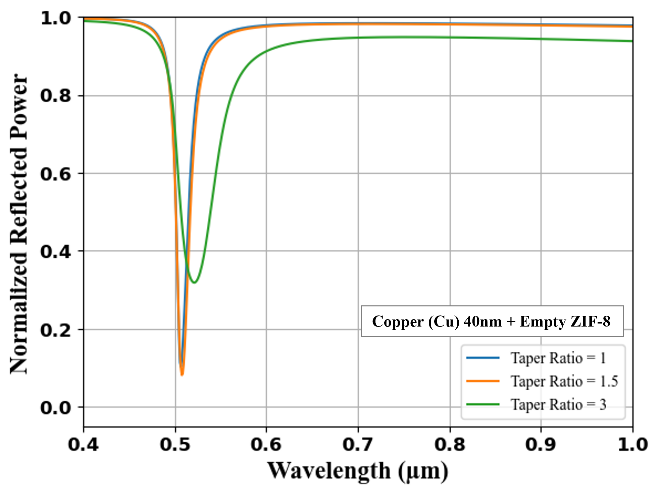


(g)

Figure S6: Optimization and comparison of plasmonic metal layers for the proposed FO-SPR sensor with an empty ZIF-8 sensing layer. (a–c) Optimization of metal layer thickness for (a) Au, (b) Ag, and (c) Cu by evaluating the SPR response for different metal thicknesses. (d) Comparison of the optimized SPR spectra of Au, Ag, and Cu for the taper ratio of 1. (e–g) SPR responses of the empty ZIF-8 configuration for taper ratios of 1, 1.5, and 3 using the optimized metal thicknesses of (e) Au, (f) Ag, and (g) Cu, respectively.

**S3. Proof-of-concept Experiment**


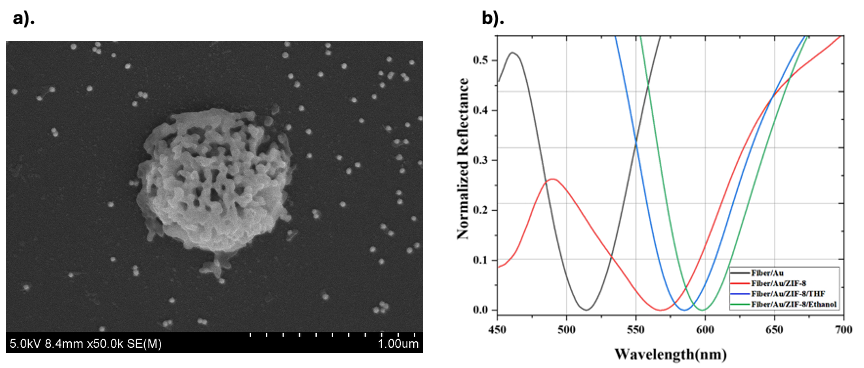


**Fig S7.** (a) SEM micrographs of ZIF-8 grown over Au immobilized fiber tip probe. (b) Normalized reflectance spectra showing the optical response of the sensor at different fabrication and sensing stages. The initial growth (black) and ZIF-8 thin-film deposition (red) establish the baseline resonance. Subsequent exposure to tetrahydrofuran (THF, blue) and ethanol (green) results in a significant redshift of the reflectance minimum, demonstrating the sensor's refractive index sensitivity.

| TAPER RATIO | SENSITIVITY BY VARYING VOLUME FILL FACTOR OF GUEST MOLECULE  (nm/Volume factor) | |
| --- | --- | --- |
|  | THF (at 40%) | DMF (at 40%) |
| 1 | 9.3795 | 4.8284 |
| 1.5 | 9.6093 | 4.9577 |
| 3 | 10.7569 | 5.6988 |

S1 – Table: sensitivity values of different guest molecules

Table S2: Different metal optimization of various taper ratios

| Taper Ratio | COPPER (Cu) | | | SILVER (Ag) | | | GOLD (Au) | | |
| --- | --- | --- | --- | --- | --- | --- | --- | --- | --- |
|  | SENSITIVITY (nm/RIU) | FWHM (nm) | FOM (RIU^-1^) | SENSITIVITY (nm/RIU) | FWHM (nm) | FOM (RIU^-1^) | SENSITIVITY (nm/RIU) | FWHM (nm) | FOM (RIU^-1^) |
| 1 | 2500 | 15.1 | 165.56 | 2600 | 44.6 | 58.29 | 3000 | 130.3 | 23.02 |
| 1.5 | 2600 | 16.7 | 155.68 | 2700 | 46.9 | 57.56 | 3100 | 134.8 | 22.99 |
| 3 | 2800 | 44.6 | 62.78 | 3000 | 76.8 | 39.06 | 3600 | 196.3 | 18.33 |

Table S3: sensitivities of different taper ratios

| SENSITIVITY | | | | |
| --- | --- | --- | --- | --- |
| TAPER RATIO | BY VARYING VOLUME FILL FACTOR OF GUEST MOLECULE  (nm/Volume factor) | | BY VARYING RI  (nm/RIU) | |
|  | THF (at 40%) | DMF (at 40%) | THF (when  Δn= 0.01) | ETHANOL (when Δn= 0.01) |
| 1 | 9.3795 | 4.8284 | 4000 | 6300 |
| 1.5 | 9.6093 | 4.9577 | 4200 | 6600 |
| 3 | 10.7569 | 5.6988 | 5300 | 9900 |
